# MicroRNA: Implications for Alzheimer Disease and other Human CNS Disorders

**DOI:** 10.2174/138920209788185252

**Published:** 2009-05

**Authors:** Olivier C Maes, Howard M Chertkow, Eugenia Wang, Hyman M Schipper

**Affiliations:** 1 Bloomfield Centre for Research in Aging, Lady Davis Institute for Medical Research, Sir Mortimer B. Davis Jewish General Hospital, Montreal, Canada; 2 Departments of Neurology & Neurosurgery and Medicine (Geriatrics), McGill University, H3T1E2, Montreal, Canada; 3 Gheens Center on Aging & Department of Biochemistry and Molecular Biology, School of Medicine, University of Louisville, Louisville, Kentucky 40202, USA

**Keywords:** Aging, Alzheimer disease, biomarker, microRNA, mild cognitive impairment, neurodegeneration, genomics.

## Abstract

Understanding complex diseases such as sporadic Alzheimer disease (AD) has been a major challenge. Unlike the familial forms of AD, the genetic and environmental risks factors identified for sporadic AD are extensive. MicroRNAs are one of the major noncoding RNAs that function as negative regulators to silence or suppress gene expression *via* translational inhibition or message degradation. Their discovery has evoked great excitement in biomedical research for their promise as potential disease biomarkers and therapeutic targets. Key microRNAs have been identified as essential for a variety of cellular events including cell lineage determination, proliferation, apoptosis, DNA repair, and cytoskeletal organization; most, if not all, acting to fine-tune gene expression at the post-transcriptional level in a host of cellular signaling networks. Dysfunctional microRNA-mediated regulation has been implicated in the pathogenesis of many disease states. Here, the current understanding of the role of miRNAs in the central nervous system is reviewed with emphasis on their impact on the etiopathogenesis of sporadic AD.

## INTRODUCTION

### 1. The Pathology and Epidemiology of Alzheimer Disease

Alzheimer disease (AD) is an aging-associated dementing disorder characterized by progressive neuronal degeneration, gliosis, and the accumulation of extracellular deposits of amyloid (senile plaques; SP) and intracellular inclusions (neurofibrillary tangles; NFT) in discrete regions of the basal forebrain, hippocampus, and association cortices [1, 2]. Rare early-onset familial forms of AD with an inheritable autosomal dominant mutation in either amyloid precursor protein (APP), presenilin-1 (PSEN1) or presenilin-2 (PSEN2) genes, account for <1% of AD cases. An unknown proportion of late-onset familial AD harbors “less aggressive” familial autosomal dominant mutations [3, 4]. The vast majority of AD cases are sporadic, with complex genetic, environmental and epigenetic risk factors [3, 5-7].

Susceptible genetic polymorphisms in over 500 genes have been proposed as risk alleles [8], but only the apolipoprotein E (APOE) ε4 allele has been validated [9-11]. In these subgroups, the APOEε4 allele acting with other genetic polymorphisms accelerates disease onset. There thus appear to exist multiple etiopathogenetic pathways which culminate in a common histopathological profile characteristic of sporadic AD. A sub-classification taking into account AD family history, age of onset, ethnicity, risk factors (i.e. education, lifestyle, vascular pathology) and possibly gender could facilitate the delineation of discrete at-risk genetic networks associated with AD.

Definitive AD diagnosis is established by the postmortem quantification of SP and NFT in affected brain regions. The amyloid and neuritic plaques arise from the accumulation of 1-40 and 1-42 amyloid beta (Aβ) peptide residues following the sequential cleavage of the APP protein by β-secretase (β-site APP cleaving enzyme 1, BACE1) and γ-secretase complex (PSEN1 and PSEN2) [12]. Increases in Aβ_42_ oligomers are believed to contribute to synaptic failure, oxidative stress and NFT [13]. NFT are filaments composed of a hyperphosphorylated microtubule-associated protein, tau [14].

The increases in Aβ and SP as well as NFT in the brain are aging-related phenomena and occur in the brains of the non-demented elderly. In familial AD, the ratio of Aβ_42_/Aβ_40 _specifically increases [15]. However, mutations in the presenilins and APP genes may also alter their signaling [16, 17] or transactivation functions [18, 19]. In sporadic AD, different mechanisms which alter either Aβ production or clearance have been identified. For example, increases in BACE1 expression and protein in AD brains correlate with higher Aβ production [20]. Alternatively, APP cleavage by cathepsin B followed by glutaminyl cyclase modification of the truncated Aβ (3–42) into pyroglutamate (pE)-modified Aβ_3(pE)–42_ may also contribute to AD pathology in other subgroups [21, 22]. Likewise, multiple pathways have been suggested to cause the polymerization of NFT, including Aβ_42_ [13], caspase activation [23] or decrease in insulin-like growth factor I (IGF-I) signaling [24].

Several age-related disorders that contribute to AD pathology have been described, in particular dysfunctions in antioxidant responses and mitochondrial homeostasis [25-27]. Higher oxidative damage in both peripheral and CNS tissues has been documented in AD patients, even at early stages of the disease [28]. Other abnormalities associated with AD include impairment in DNA repair [29], deregulation of calcium homeostasis and altered immune responses [30], as well as evidence of neuroinflammation [31, 32] and cerebrovascular pathology [33]. In many cases, oxidative stress is a common denominator and could be linked to underlying genetic hubs shared by these pathways. We recently identified an impaired genetic expression network in sporadic AD that may coordinate oxidative stress defense mechanisms and DNA repair responses [9].

Considerable efforts in defining surrogate biomarkers in order to facilitate the diagnosis of AD are underway (reviewed in [34]). The most robust AD biomarker at this time is the combined decrease in Aβ_42_ and increase in phosphorylated tau levels in cerebrospinal fluid (CSF). Unfortunately, this combination of biomarkers is not useful for the prediction of earlier stages without clinically defined dementia, such as mild cognitive impairment (MCI) [35]. Moreover, lumbar puncture for CSF examination is relatively invasive, and unsuitable for mass screening of the aging population. Readily accessible biomarkers from a minimally-invasive blood (or other peripheral) specimen would greatly facilitate the management of this condition [34].

Impairment in miRNA expression or function contributes to human diseases and has great potential as disease biomarkers [36]. Although their modes of action are complex, miRNAs generally act as post-transcriptional repressors of their target gene and/or protein [37, 38]. Major implications for miRNAs in the etiological pathways and associated risk factors may be anticipated in AD. Characterization of underlying etiopathogenetic pathways related to miRNA dysfunction holds promise for the diagnosis, prognosis and management of AD and other neurological diseases. We provided evidence of augmented miRNA expression in peripheral blood mononuclear cells (PBMC) in sporadic AD, which may shed new light on the pathogenesis of AD and provide accessible blood-based diagnostic and prognostic biomarkers for this condition [39].

## THE ROLES OF MICRORNA IN THE CENTRAL NERVOUS SYSTEM

### 1. Small Noncoding MicroRNAs

The unprecedented discovery of small noncoding miRNAs and their implications for virtually all biological functions [40-42] is still in early stages of research with at least 1000 or more miRNAs expected to be identified in the human genome [43]. At present, 706 human miRNA genes have been registered in stem-loop form in the web-database, miRBase (Sanger Institute, version 13.0) [44]. With the exception of the founding let-7 family of microRNAs in mammals [45], their annotation is based on their cloning chronology, with family members distinguished in alphabetical and/or numerical order [46].

The great majority of miRNA genes are transcribed by RNA polymerase II under the regulation of type II promoters, with a few exceptions being constitutively expressed by type III promoters [47]. MicroRNAs are found in various inter- and intragenic locations, and at least 42% are estimated to be expressed in clusters [48]. The pri-miRNA transcripts are cleaved by the endonuclease Drosha complex into hairpin secondary structures called pre-miRNAs in the nucleus, and then exported into the cytoplasm where they are cleaved by the endonuclease Dicer into 18 to 22 nucleotide long mature miRNAs [49]. MicroRNA expression is regulated not only at their promoter, but also at post-transcriptional levels of processing [50]. In fact, Dicer is localized to the somatodendritic compartment in neurons and glia [51] and is activated to process pre-miRNA precursors following synaptic signaling [52].

The mature miRNAs are then assembled into RNA-induced silencing complexes (RISC) and bind to partially complementary sites mostly within the 3' untranslated region (UTR) of target mRNAs (reviewed in [49, 53, 54-56]). Regulatory subunits within the RISC complex and/or present on the mRNAs (i.e. cytoplasmic ribonucleoprotein complexes, mRNPs) play decisive roles in miRNA-mRNA localization, miRNA responsiveness to cellular signaling and mode of action [37, 56, 57]. The miRNP-mRNA complex may be directed to polyribosomes in sub-cellular compartments, where miRNA regulate translation. Alternatively, the complex accumulates in foci termed P-bodies and/or granules for mRNA storage or degradation [53, 56]. While the various regulatory factors and signaling effectors associated with miRNA regulation are not fully understood, an example of a stress responsive mechanism to redirect stored mRNA in P-bodies back into translation has been described for the RNA-binding protein, HuR [58]. Similar mechanisms of reversal in miRNA-mediated repressions are expected within miRNP-mRNA complexes in diverse brain and synaptic functions [56].

The principles of target recognition for miRNA:mRNA duplex formation are based on the complementarity of an eight nucleotide seed region (reviewed in [59-61]). Various algorithms are now available to predict major evolutionarily conserved targets in 3'UTR for over 60% of mRNAs [61]. One single miRNA has hundreds of potential gene targets, whereas dozens or more microRNA response elements (MRE) are predicted in the 3'UTR of individual transcripts. However, mechanisms of alternative splicing [62] and 3'UTR cleavage [63] have evolved to alter miRNA regulation in specific cellular context. Improvement of target prediction by algorithms are expected to eventually take into account such variables as cell-specific splicing, MRE accessibility within mRNA secondary structures [64], the presence of protein binding sites [65], and perhaps RNA editing [66, 67]. In fact, miRNA editing occurs at a high frequency in the brain and may serve to silence miRNAs [68]. Alternatively, miRNA editing may also reinforce and stabilize miR:mRNA duplex in order to favor target silencing by mRNA cleavage [69].

Multiple miRNAs are involved in regulating either a single gene or sets of genes in common and divergent genetic networks. Despite the extreme level of complexity in miRNA regulatory networks, one approach to facilitate the identification of biological functions is to integrate genomic (i.e. mRNA, miRNA) and proteomic expression data with miRNA target predictions in the same cellular context. This approach successfully identified target predictions from various algorithms in various species and experimental systems, some of which are validated in cell-based functional assays (Table **1A, 1B**). Eventually, bioinformatics tools will assist in the delineation of regulatory networks and feedback loops, by integrating signaling and transcription factors and identifying the presence of regulatory elements and links with other miRNAs. Despite the arduous task ahead, key miRNAs and their regulatory feedback loops have been identified with specific CNS functions and neurological disorders (Table **1A, B**).

### 2. The Identification of MicroRNAs in the CNS

The role of miRNAs in neuronal development and function was first elucidated in *Caenorhabditis elegans* [110]. The importance of miRNAs in CNS functions and disease is now well recognized (reviewed in [111-122]). Initially, the implication of miRNAs in mammalian CNS was determined by miRNA expression profiling [117, 123]. Among the first hundred miRNAs cloned, miR-7, -9/9*, -124a, -124b, -125a, -125b, -128, -132, -135, -137, -139, -153, -149, -183, and miR-219 were reported to be abundantly expressed in mouse and human adult brains [124]. Several more miRNAs, including let-7 family members were shown to co-purify with polysomes in neurons [125]. Similar miRNAs were also identified in rat and monkey [126].

More refined cellular specificity in their expression was reported for astrocytes (miR-23, -26, -29), neurons (miR-124, -128) [127], and hippocampal neurons (let-7 family) [50]. Cellular speciation associated with orthologue miRNAs was also reported in zebra fish for neuronal precursors (miR-92b), mature neurons (miR-124, -181, -222), motor neurons (miR-218a) and dendrites (miR-134) [128]. Likewise, in rat hippocampal neurons, miRNAs have been sub-localized within soma and dendrite compartments, including miR-124 and miR-26a respectively [79]. In human prefrontal cortex, miR-30a was shown to be enriched in pyramidal neurons [80]. A detailed comparison of miRNA expression profiles in mouse and zebra fish CNS revealed species differences [129]. Eventually, a tissue and sub-cellular cartography of microRNA levels in human CNS should contribute to a more complete understanding of their functions.

A detailed functional analysis for a human miRNA was accomplished for the predominant brain miRNA, miR-124 [130]. Direct evidence of a role for miR-124 was derived from its over-expression in HeLa cells, which shifted the gene expression pattern to that of a neuron [131]. Expression of miR-124a was shown to be under the negative control of the transcriptional repressor, REST (RE1 silencing transcription factor), a regulator of neuronal differentiation [132]. In turn, miR-124 targets the REST-binding anti-neural factor, SCP1 (Small C-terminal domain phosphatase 1), and reinforces the induction of neurogenesis [81]. Its constant expression also maintains neuronal speciation [82], in part by targeting the polypyrimidine tract binding protein 1 (PTBP1/hnRNPI), a repressor of alternative splicing expressed in non-neuronal cells [83]. Thus, miR-124 may act at different levels of gene regulation for neuronal speciation, involving complex regulatory and splicing mechanisms and negative feedback loops [133].

Another key miRNA under the regulation of REST is miR-9 [134]. This miRNA is reported to have several functions depending on its cellular context, such as in CNS development [71], midbrain-hindbrain boundary formation [75], or in oxidative stress responses [107]. In Drosophila, miR-9 regulates the transcription factor *Senseless* in the development of the peripheral nervous system [76]. MicroRNA miR-9 is also required for brain functions, as suggested in PSEN1 null mice, where its decrease is associated with severe brain defects [72]. The loss of PSEN1 impacts memory, synaptic plasticity and induces neurodegenerative changes [135, 136].

Also noteworthy is the let-7 family which is strongly expressed in brain and neuronal stem cells [50]. The evolutionarily conserved let-7 family members are highly edited [69], and the processing of pri-let-7 into active mature miRNAs is tightly regulated in the brain [50]. One possible post-transcriptional regulation is by the RNA binding protein, Lin28 [137], which binds to the pri-let-7 precursor in order to inhibit its processing by Drosha [138, 139]. The expression of miR-125 during neurogenesis suppresses Lin28 [84] and together with let-7 further represses Lin28 in an autoregulatory circuit [140]. Moreover, let-7 directly regulates Dicer and may have a global regulatory control on miRNA functions [141]. At this time, no specific CNS functions are described for the let-7 family members in humans; however, their roles in differentiation and as tumor suppressors have been demonstrated [142].

### 3. MicroRNA Associated with Neurological Diseases

The relationship between microRNA dysfunctions and neurological diseases is perhaps best illustrated with fragile X mental retardation. In these patients, the absence of Fragile X mental retardation 1 protein (FMRP) impairs Dicer and RISC functions required for miRNA-mediated synaptic plasticity and dendritic development [143]. Experimentally, altering miRNA processing by the inactivation of Dicer in Drosophila also prevents dendritic development [144] and enhances polyglutamine and tau-induced neurodegeneration [145]. Likewise, inactivation of Dicer in the CNS of mice impairs dendrite formation, neuronal survival, and gradually leads to neurodegeneration [146-148].

Dysfunction in RISC complex formation also occurs in the pathology of Huntington disease (HD). The interaction of the HD protein with one of the RISC subunit Argonaute-2 is impaired in HD, which prevents the formation of P-bodies [149]. Moreover, impairment in HD protein regulation of REST results in the repression of miRNA expression in HD brains, in particular the down-regulation of miR-132 affecting neurite outgrowth [101] and the down regulation of miR-124a and miR-9/9* affecting a double negative feedback loop regulation of REST with miR-9 [100]. This suggests that the down-regulation of key miRNAs regulated by REST predisposes to neurodegenerative disorders.

The association of miRNA with another polyglutamine disease, dentatorubral-pallidoluysian atrophy, is suggested in Drosophila; mutation in miR-8 (orthologue miR-200a/b and miR-429) causes apoptosis in the brain and behavioral defects, along with an increase histone deacetylase (HDAC) activity [70]. HDAC inhibitors have protective effects in the treatment of stroke, AD, amyotrophic lateral sclerosis (ALS) and HD [150] and alter both gene and miRNA expression [151]. This suggests that miRNAs such as the miR-200 family could be involved in HDAC deregulation in neurological diseases.

Interestingly, miR-200a was found to be increased in AD brain [99] and peripheral blood mononuclear cells (PBMC) [39]; however, its function in AD pathology is not yet established. A specific role has been described for miR-200b in the regulation of a ZFH-1 repressor (ZFHX1B), involved in the regulation of E-cadherin expression in mouse brain [89]. Importantly, E-cadherin is a substrate of PSEN1, which is related to diverse brain functions and synaptic plasticity [152]. The miR-200 family is also involved in functions of the olfactory system [88], pathways implicated in neurodegenerative conditions such as AD and Parkinson disease (PD) [153].

In PD, miR-133b is deficient in midbrain dopaminergic neurons, and may be related to an impaired feedback loop with the Paired-like homeodomain transcription factor, PITX3 [103]. Polymorphisms in PITX3 are associated with PD [154], and perhaps this impairs its capacity to induce miR-133b expression. In another study, a polymorphism associated with PD modified the microRNA binding site of miR-433 in the transcript of fibroblast growth factor 20 (FGF20), resulting in increased alpha-synuclein expression [155]. Other polymorphisms have been associated with neurological disease, such as in the noncoding genes of miR-198 and miR-206 associated with schizophrenia [156], as well as in the binding site of miR-189 in Slit and Trk-like 1 (SLITRK1) mRNA associated with Tourette syndrome [157].

Genetic defects such as trisomy 21 (Down syndrome, DS) lead to neurodegenerative pathology consistent with AD. In particular, extra copies of APP [158] and Dyrk1a [159] genes on chromosome 21 contribute, respectively, to SP and NFT, lesions typical of AD-affected brain. Moreover, extra copies of let-7c, miR-99a, -125b-2, -155, and miR-802 on chromosome 21 are also over-expressed in DS patients [106]. Interestingly, increased Dyrk1a levels may augment REST expression [160], suggesting that other microRNAs under the regulation of REST could be affected.

## MICRORNAs AND ALZHEIMER DISEASE PATHWAYS

### 1. MicroRNA Profiling in Alzheimer Disease

The entorhinal cortex-hippocampus system, concerned with memory functions, is among the first regions of the brain to be affected by AD pathology [161, 162]. In an initial expression profiling of the 13 most abundant brain miRNAs mentioned above [124], Lukiw [98] reported the up-regulation of miR-9, miR-125b and miR-128 in AD affected hippocampus (Table **1B**). Later, 328 miRNA were profiled in the cortex of sporadic AD, showing both up- and down-regulation in miRNAs [96]. Among over 400 miRNAs profiled, several up-regulated miRNA were reported in AD-affected hippocampus and medial frontal gyrus, cerebellum and CSF [99]. Likewise, we detected mostly an up-regulation of miRNA expression in PBMC of sporadic AD as compared to aged-matched normal elderly control subjects [39].

The up-regulation of miR-125b and down-regulation of miR-9 and miR-210 have been consistently reported in different studies on miRNA expression profiling of AD-affected brain (Table **2**). The deregulation of miR-125b and miR-9 in AD is particularly interesting because, as described above, these key miRNAs are involved in brain development and function as well as in neurological diseases such as DS and HD. The up-regulation of miR-197 and down-regulation of miR-15, -146b, -181c, and miR-338 are commonly altered in AD brain parenchyma and CSF.

**Fig. (1):**
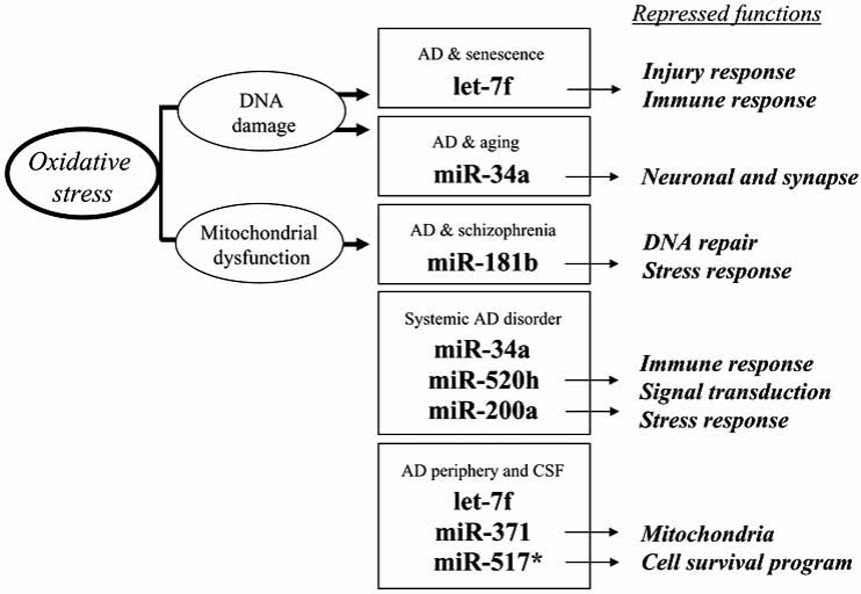
Up-regulated microRNA in peripheral blood mononuclear cells (PBMC) of sporadic Alzheimer disease (AD) patients. MicroRNAs are small noncoding RNA which generally act as post-transcriptional repressors of gene and/or protein expression. Their common up-regulation in AD brain, cerebrospinal fluid (CSF) and PBMC as well as in other neurological disorders and aging model systems is shown, with implications for oxidative stress, DNA damage and mitochondrial dysfunction. Down-regulated genes in Alzheimer PBMC [163] which may be repressed by these miRNAs were identified using target predictions from miRanda algorithm [44]. Major functional categories which may be theoretically repressed in AD-affected PBMC are indicated.

MicroRNAs similarly expressed in both AD-affected brain and PBMC are identified (Table **2**; Fig. **1**), including the up-regulation of miR-34a, miR-200a and miR-520h, as well as the up-regulation let-7f, miR-371 and miR-517/517* observed in the CSF and PBMC of AD patients. Of note, miR-520h is reported to be highly up-regulated in AD brain [96], and its increase in Alzheimer PBMC suggests it may have a major role in the systemic manifestations of the disease. Systemic dysfunction is also indicated by the up-regulation of miR-34a and miR-200a in AD cortex and PBMC.

We suggest here that the systemic increase in specific miRNAs may suppress various cellular functions in AD, such as redox defenses or DNA repair mechanisms, by targeting mRNA and/or protein species in brain and peripheral tissues (Fig. **1**). Indeed, gene expression studies in AD have shown substantial down-regulation of various mRNA species in brain [164], PBMC [163] and lymphocytes [165] relative to non-demented controls. Likewise, impairment in protein synthesis is known to occur in AD [166] leading to diminished protein levels in the CSF [167] and plasma [168] of these patients.

For example, the up-regulation of peripheral miRNAs in AD could contribute to the diminished plasma proteins reported to be predictive biomarkers for AD [168], such as chemokine-7 (let-7f, miR-181b) and interleukin-1α (miR-181b, -200a). Moreover, in our genomics work on Alzheimer PBMC, we observed on one hand a significant number of down-regulated genes [163], while on the other, only up-regulation of miRNAs was detected [39]. Interestingly, predicted targets for up-regulated miRNAs correlated with down-regulated genes in functional categories of Synapse Activity, Transcription, and Injury/Redox Homeostasis and DNA Damage (Fig. **1**). This suggests that the systemic up-regulation of miRNAs in AD may contribute not only to impaired redox homeostasis and DNA repair mechanisms, but also reflect homologous CNS synaptic dysfunction occurring in AD brains. Importantly, this supports the notion that peripheral miRNA, gene and protein expressions may serve as diagnostic and prognostic biomarkers, and possibly provide leads to the development of new therapeutic interventions.

### 2. MicroRNA Associated with AD Etiopathogenesis

Although disorders of APP cleavage have been implicated in the etiopathogenesis of AD, experience with Down syndrome (DS) raised the possibility that hyper-expression of the APP gene may contribute to Aβ production in some forms of sporadic AD. In this respect, there is a long list of predicted miRNAs (i.e. from DIANA-microT, miRanda, TargetScanS algorithms) that may target the 3'UTR of APP, including let-7i, miR-15, -26, -29, -93, -101, -106, and miR-181 which are reportedly down-regulated in AD brain [96, 99].

**Fig. (2):**
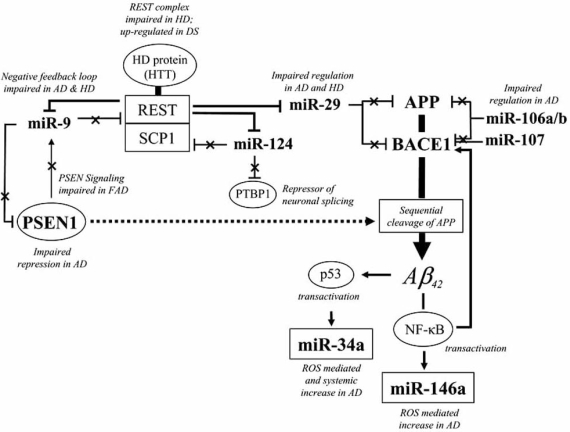
A simplified scheme of etiopathogenetic pathways associated with microRNA dyregulation in sporadic Alzheimer disease (AD). Sporadic AD may be characterized by the increase in hallmark genes (i.e. APP, BACE1, PSEN1) which contribute to amyloid beta (Aβ) production by sequential cleavage of APP. Altered miRNAs reported in AD brain which target these genes (as predicted by miRanda and TargetScan algorithms and validated in cell-based assays) are shown [93, 94, 96, 97, 108]. The REST complex negatively regulates the expression of miR-9, miR-29 and miR-124 [134]. The negative feedback loop by miR-9 which suppresses REST is impaired in AD and other neurological diseases. Familial AD (FAD) PSEN1 loss-of-function mutations are recapitulated by PSEN1 knock-out in mouse [72], leading to the decrease in miR-9 akin to that observed in sporadic AD brain. Oxidative stress and DNA damage, generated in part by elevations in amyloid beta (Aβ), iron and reactive oxygen species, induce miRNA expression as exemplified by miR-34a and miR-146a. The stress responsive transcription factor p53, which may increase in AD by Aβ transactivation [173], may further transactivate miR-34a [174]. Stress-mediated neuroinflammatory responses are represented by nuclear factor-kappa B (NF-κB) which transactivates the expression of miR-146a [108] and BACE1 [175]. Barred lines denote post-transcriptional repression of target genes and crossed lines denote de-repression associated with the down-regulation of miRNAs.

In *C. elegans*, the APP-like orthologue is targeted by the single let-7 supporting the notion that the let-7 family of miRNAs in humans could be involved in the regulation of APP [92], e.g. let-7i which is reported to decrease in AD brain [96]. In humans, the over-expression of miR-106a/b was shown to reduce APP protein levels in kidney cells [93, 94]. This validates this target prediction for APP, and is consistent with the fact that miR-106a/b is down-regulated in AD-affected brain [94]. The down-regulation of miR-106a/b (Fig. **2**), perhaps with other down-regulated miRNAs (let-7i, miR-15, -26, -29, -93 -101), may favor higher APP levels in AD brains.

The Aβ cleavage product of APP may then arise from the increase in BACE1 and PSEN1 levels (Fig. **2**). Of note, BACE1 and APP appear to be targeted by similar sets of miRNAs, including miR-9, -15, -27, -29, and miR-101. These miRNAs may therefore facilitate increases in both APP and BACE1 in AD brain. In fact, over-expression of miR-29a/b-1 reduces APP and BACE1 levels in kidney cells [96]. Another miRNA that targets BACE1 and is down-regulated in early AD and MCI-affected cortex is miR-107 [97]. In this latter example, the identification of miRNA binding sites in BACE1 was determined without stringent cross-genome conservations using the algorithm *rna22* (as opposed to miRanda or TargetScan used above).

Mutations in PSEN1 and PSEN2 account for the majority of FAD cases and the products of these genes have been implicated in sporadic AD as well [135]. In PSEN1 knock-out mice, Notch signaling and transcriptional networks associated with miR-9 expression decrease and reflect to some extent the loss-of-function PSEN1 mutations associated with FAD pathology [72]. In the pathogenesis of sporadic AD, augmented PSEN1 mRNA levels [171, 172] may foster Aβ production by sequential cleavage of APP (Fig. **2**). Interestingly, miR-9 is predicted to target PSEN1, suggesting that the up-regulation of PSEN1 could be associated with the decline in miR-9 levels in AD.

The down-regulation of miR-9 and miR-29 is, however, not specific to AD (Table **1B**) as these miRNA species are also diminished in the brains of individuals with schizophrenia [105] and HD [100]. Conceivably, aberrant profiles of neural miRNA expression shared by various neurological disorders may give rise to common patterns of cellular dysfunction in these conditions. Moreover, deregulations in gene and protein expression may also include other mechanisms, such as long noncoding antisense BACE1 transcript which increases BACE1 expression in AD [176]. Thus, alterations in noncoding RNA expression which affect CNS functions or are associated with neurological diseases are not limited to miRNA species (review in [177]).

### 3. MicroRNA Associated with Oxidative Stress and AD Risk Factors

Aging remains the most robust risk factor thus far identified for sporadic AD and other human neurodegenerative conditions. Mechanisms of aging under the regulation of miRNAs may contribute to AD pathology, such as those identified in various aging model systems, including the up-regulation of let-7f, miR-30d, -34a, -432, -517 and down-regulation of let-7i and miR-451 (Table **2**). These miRNA may reflect intrinsic aging mechanisms in AD [178]. In particular, there is a systemic increase in miR-34a in AD brain [99] and PBMC [39], as well as in aging mouse liver [169] and *C. elegans* [170]. The up-regulation miR-34a may partly result from DNA damage [179], which accumulates during aging and to an even greater extent in AD [180]. Likewise, the increase in let-7f may be related to DNA damage and cellular senescence, as it is up-regulated in replicatively senescent cells [181] and Alzheimer CSF [99] and PBMC [39]. Replicative senescence correlates with gradual telomere shortening and DNA damage, largely due to the high oxygen tension (20% O_2_) that exists in culture [182]. Importantly, the up-regulation of let-7 in DS and miR-34a in AD (Table **1B**) also suggests that mechanisms of aging and senescence, perhaps related to DNA damage responses, are involved in degenerative processes within the brain.

Oxidative DNA damage is an early and systemic event in the pathophysiology of AD [183, 184]. In our studies on miRNA and gene expression in AD PBMC, we observed impaired DNA repair and antioxidant gene responses, which correlate with the up-regulation of miR-181b, -200a, -517* and miR-520h, and may possibly repress DNA repair and oxidative stress response mechanisms [39]. Oxidative damage in AD tissues also accrues from a gradual decline in antioxidant defenses and increasing neural levels of redox-active transition metals and other pro-oxidant species [26]. AD-associated miRNAs activated by stress-inducing agents, such as aluminum and iron sulfates, include miR-9, miR-125b and miR-128 [98, 107].

Another major source of reactive oxygen species in AD stems from infidelity of electron transport in dysfunctional mitochondria [26] which can be recapitulated using arsenic and other stressors [185, 186]. Similar to miRNA deregulation in AD, human lymphoblastoid cells up-regulate miR-34a and down-regulate miR-210 following arsenic treatment, as well as up-regulate miR-125b, -130, -145 and miR-181b under conditions of folate deficiency [187]. Folate deficiency may cause cognitive impairment and decrease DNA repair mechanisms with age [188, 189]. Analogous patterns of altered miRNA expression in AD suggest that similar mitochondrial and ROS mediated dysfuntions are associated with these miRNAs. Interestingly, miR-181b is up-regulated in schizophrenia-affected brains where mitochondrial dysfunction has been documented [190], and may warrant further investigation in AD brain in light of its up-regulation in AD PBMC.

Stress-responsive miRNAs that are implicated in cardiac hypertrophy [191] and vascular disease [192] may similarly participate in the vascular pathology of AD, pure vascular dementia and mixed dementia [193, 194]. In this context, prime examples of the latter include the up-regulation of miR-27a/b [99], miR-125b [98] and down-regulation of miR-93 [96], as observed in AD brain. It may be of particular interest to determine whether the over-expression of miR-155 in DS brain is associated with macrophage-related inflammation [195]. Also characteristic of AD-affected neural tissues are neuroinflammatory responses activated by the transcription factor, nuclear factor-kappa B (NF-κB), which induces the expression of miR-146a [108]. The expression of miR-146a was also shown to be induced by ROS-generating agents such as hydrogen peroxide and Aβ_42_. Taken together, these data indicate that up-regulation of miR-27a/b, -125b, -146a and down-regulation of miR-93 may contribute to the stress-related vascular and inflammatory components of AD pathology.

## PERSPECTIVE

Deregulation of miRNAs in the CNS may impact a wide range of cellular functions that are not limited to the etiopathogenesis of AD. The identification of homologous patterns of miRNA expression among diverse neurological conditions may denote common pathways of neuronal dysfunction and degeneration. For example, the up-regulation of miR-181b in schizophrenia, or let-7 and miR-125b in DS, are also observed in sporadic AD and may denote common mechanisms of disease (Table **1B**). If confirmed, single pharmaceuticals targeting specific miRNA regulatory pathways may prove effective in the treatment of multiple neurological afflictions. The probable decline of miR-9 in AD suggests that its target REST may induce the repression of other neuronal genes and miRNAs (i.e. miR-29a/b, -124, and -132) as reported in the hereditary neurodegenerative disorder, HD [100].

Discrepancies in miRNA expression data in AD are not unexpected and further studies will be required to determine whether disparate data are due to genetic or demographic determinants. Genetic polymorphisms in miRNA sequences, in their promoter regions or in the 3'UTR binding sites of their targets may alter miRNA expression and function. Different genetic backgrounds among familial or sporadic AD patients could well be reflected by specific miRNA expression signatures. For example, we found more robust up-regulation of miR-181b and miR-371 in Alzheimer PBMC relative to control subjects in the APOEε4-positive stratum [39]. This suggests that a higher expression of specific miRNAs in APOE4-positive carriers may contribute to earlier onset of the disease as observed in these patients. It also raises the possibility that specific PBMC gene and miRNA expression signatures may distinguish the various at-risk genetic backgrounds associated with AD.

To understand more thoroughly the role of miRNAs in diseases of the CNS, target predictions will need to be refined taking into account variables such as RNA editing, splicing and secondary structures. The elucidation of miRNA’s role in AD pathology is based on their expression in affected brain and on predictions of their targets, the latter being validated in cell-based functional assays. The fine tuning of a gene (or protein) level is not limited to one miRNA [196], and the net effect on a given target shared by concomitantly down- and up-regulated miRNAs remains unclear. The identification of up-regulated miRNAs having targets which nonetheless increase in the same AD brain tissue (i.e. APP, BACE1) may suggest loss-of-function either by genetic polymorphisms or miRNA editing.

At present, the impact of miRNA deregulation in complex regulatory networks within the brain is not known, nor how sets of miRNAs interact in the regulation of shared targets. To delineate further salient cause-effect relationships in these regulatory pathways, more in-depth characterization of the promoter elements and various transcription factors and repressors (i.e. REST, Aβ, NF-κB, p53, etc.) which alter miRNA expression is paramount. It will also be necessary to determine which sets of miRNAs are affected in different genetic backgrounds and regions of the CNS, and how they integrate within the various regulatory loops and diverging networks implicated in AD and other neurological diseases.

Future studies employing relevant transgenic mouse models should facilitate the elucidation of the beneficial or detrimental effects of specific sets of miRNA in AD pathology. As systemic oxidative damage is characteristic of the AD phenotype, the silencing of specific up-regulated miRNAs (i.e. miR-34a, -181b, -520h) which may impair stress defense and repair mechanisms could reduce ROS levels and related cellular dysfunctions in AD. Conceivably, specific sets of miRNAs will one day be used as a disease-modifying treatment of AD [197]. In the inexorable progress towards personalized medicine, the design of therapeutic miRNA regimens to modify diseased gene expression profiles may be informed by highly-individualized genomic signatures. It is also conceivable that miRNAs may provide minimally-invasive, blood-based biomarkers to facilitate early diagnosis and prognosis of MCI and AD and serve as surrogate indices of successful therapeutic interventions in these and other human CNS afflictions [198].

## Figures and Tables

**Table 1A:** Identification of MicroRNA Associated with the Central Nervous System

MicroRNA	Cell/Tissue	Target^1^.	Functions	Sp.	Ref.^2^.
*Down-Regulated*					
miR-9, 131	Presenilin 1 knockout	-	Brain development	m	[72]
miR-130a, 206	Mesenchymal stem cell	TAC1	Neurotransmission	h	[73]
*Up-Regulated*					
let-7a/c/e	P19; embryo	-	Neuronal speciation	m	[50]
miR-7	Vasotocinergic neuron	-	Neurosecretion	z	[74]
miR-9-1	Embryo	Fgf genes	CNS development	z	[75]
miR-9a	Sensory organs	*Senseless*	Sensory neuron	d	[76]
let-7g, miR-21, 27a/b, 29a, 138	SH-SY5Y	-	Neurogenesis		[77]
miR-23	NT2	HES1	Neurogenesis	h	[78]
miR-26a	Hippocampus	MAP2	Dendrite	r	[79]
miR-30a-5p	Prefrontal Cortex	BDNF	Cortical development & function	h	[80]
miR-124	P19; neural tube	SCP1	CNS development	c/m	[81]
miR-124	Neural tube	LAMC1 ITGB1	Neuronal speciation	c	[82]
miR-124	CAD; N2a	PTBP1	Brain-specific alternative splicing; Neurogenesis	m	[83]
miR-125b	P19	Lin-28	Neurogenesis	m	[84]
miR-132	PC12; neuron	p250GAP	Neuronal plasticity	r	[85]
miR-134	Hippocampus	Limk1	Dendrite & synapse	m/r	[86]
miR-184	Cortical neurons	-	Synaptic plasticity (DNA methylation)	m	[87]
miR-200 family	Embryo	neuroD, foxg1, lfng, zfhx1	Olfactory neurogenesis	m/z	[88]
miR-200b	Hippocampus	ZFHX1B	CNS development; TGFβ-signaling	m	[89]
miR-204	Choroid plexus	TRPM3	CSF associated	m	[90]
miR-430	Embryo	-	Brain morphogenesis	z	[91]

1. Reported miRNA target’s gene symbol: APP, amyloid precursor protein; BACE1, β-site APP cleaving enzyme 1; BDNF, Brain-derived neurotrophic factor; CFH, Complement factor H; fgf, fibroblast growth factor; GRIA2, Glutamate receptor subunit; HES1, Hairy and enhancer of split 1; ITGB1, Integrin β1; LAMC1, laminin γ1; Limk1, Lim-domain-containing protein kinase; MAP2, Microtubule -associated protein 2; p250GAP, p250GTPase-activating protein; PITX3, Paired-like homeodomain transcription factor Pitx3; PTBP1, Polypyrimidine tract binding protein 1, PTB/hnRNPI; SCP1, Small C-terminal domain phosphatase 1; SLITRK1, Slit and Trk-like 1; TAC1, Tachykinin; TRPM3, Transient receptor potential cation channel M3; VSNL1, Visinin-like 1; ZFHX1B, Zinc finger E-box binding homeobox 2, ZEB2.

2. The common or significantly altered miRNAs are reported from published microarray profiling data.

**Table 1B:** Identification of MicroRNA Associated with Neurological Diseases

MicroRNA	Cell/Tissue	Target^1^.	Associated Disease	Sp.	Ref.^2^.
*Down-Regulated*					
miR-8	Adult flies	Atrophin	Behavorial defect model	d	[70]
let-7	Larval	APP-like	AD model system	c.e.	[92]
miR-106a	HEK-293	APP	AD functional assay	h	[93]
miR-106b	Cortex	APP	AD	h	[94]
miR-298, 328	Brain	BACE1	AD model system	m	[95]
miR-29a/b-1	Cortex	BACE1	AD	h	[96]
miR-107	Temporal cortex	BACE1	AD, MCI	h	[97]
let-7i, miR-9, 15a, 26b, 93, 181c, 210	Cortex	-	AD	h	[96]
miR-124b	Hippocampus	-	AD	h	[98]
miR-9, 132, 146b, 210, 212, 425	Hippocampus & FG		AD	h	[99]
miR-15b, 146b, 181c, 338	CSF	-	AD	h	[99]
miR-9/, miR9*	Cortex	REST/CoREST	HD	h	[100]
miR-29b; 124a	Cortex	-	HD	h	[100]
miR-132	Cortex	p250GAP	HD	h	[101]
miR-10b; 132, 495	Brainstem	-	PD	m	[102]
miR-133b	dopaminergic neuron	PITX3	PD	h/m	[103]
miR-338-3p; 337-3p	Brain	-	Prion disease	m	[104]
miR-26b, 29b	Prefrontal cortex	-	Schizophrenia	h	[105]
miR-9, 124a, 125b	Spinal cord	-	Spina bifida model	r	[71]
*Up-Regulated*					
let-7c, miR-99a, 125b-2, 155, 802	Fetal brain	-	DS	h	[106]
miR-197, 320, 511, 520h, 516-3	Cortex		AD	h	[96]
miR-9, 125b, 128, 130, 145	Hippocampus	-	AD/ ROS generating metals	h	[98, 107]
miR-146a	Hippocampus & cortex	CFH	AD/ Aβ & ROS inducible	h	[108]
miR-27a, 34a, 92, 145, 381, 422a, 423	Hippocampus & FG	-	AD	h	[99]
let-7f, miR-30, 125a, 197, 371, 517, 520a	CSF		AD	h	[99]
let-7f, miR-34a, 155, 181b, 200a, 371, 517*	BMC	-	AD	h	[39]
miR-29a, 330	Cortex	-	HD	h	[101]
miR-218; 132	Cortex		HD	h	[100]
let-7b, miR-146a; 128; 139-5p;320; 328; 342-3p	Brain		Prion disease	m	[104]
miR-106b	Prefrontal cortex	-	Schizophrenia	h	[105]
miR-181b	Temporal cortex	VSNL1/GRIA2	Schizophrenia	h	[109]

**Table 2:** Similarly Altered Patterns of MicroRNA Expression in Alzheimer Disease and Aging Model Systems

MicroRNA^1^	Alzheimer Disease Affected Tissue					Aging Model	Reference
	Cortex	Hippocampus	Cerebellum	CSF	PBMC		
*Down-Regulated*							
let-7i	↓					↓	[96,169]
miR-9	↓	↓	↓				[99, 96]
miR-15	↓			↓			[99, 96]
miR-146b	↓	↓	↓	↓			[99]
miR-181c	↓			↓			[99, 96]
miR-210	↓	↓					[99, 96]
miR-338	↓			↓			[99, 96]
miR-451				↓		↓	[99, 169]
*Up-Regulated*							
let-7f				↑	↑	↑	[99, 39, 181]
miR-30d				↑		↑	[99, 169]
miR-34a	↑	↑	↑		↑	↑	[39, 99, 169, 170]
miR-125b	↑	↑	↑				[99, 98]
miR-197	↑			↑			[99, 96]
miR-200a	↑				↑		[99, 39]
miR-371				↑	↑		[99, 39]
miR-517				↑	↑	↑	[99, 39]
miR-520h	↑				↑		[96, 39]

1. MicroRNAs that show similar expression trends in the various AD tissues and aging models are reported. Distinct sets of miRNAs expressed in the different systems are not shown.

## ABBREVIATIONS

Aβ: = Amyloid beta
AD: = Alzheimer disease
c: = Chick
c.e.: = Caenorhabditis elegans
CNS: = Central nervous system
CSF: = Cerebrospinal fluid
d: = Drosophila melanogaster
DS: = Down syndrome
FAD: = Familial Alzheimer disease
FG: = Frontal gyrus
h: = Human
m: = Mouse
miRNA: = MicroRNA
miRNP: = MicroRNA-cytoplasmic ribonucleoprotein complex
HD: = Huntington disease
MCI: = Mild cognitive impairment
MRE: = MicroRNA response element
NFT: = Neurofibrillary tangle
PBMC: = Peripheral blood mononuclear cell
PD: = Parkinson disease
r: = Rat
RISC: = RNA-induced silencing complex
SP: = Senile plaque
TS: = Tourette syndrome
UTR: = Untranslated region
z: = Zebrafish
